# 

**Published:** 1900-05-26

**Authors:** 


### NOTICE TO CORRESPONDENTS.

**All MS., Letters, Books for Review, and other matters intended for the Editor should be addressed The Editor, "The Hospital," The Hospital Building, 28 & 29, Southampton Street, Strand, W.C.**

**The Editor cannot undertake to return rejected MS., even when accompanied by stamped directed envelope.**

### Notice to Advertisers and Subscribers.

**All Advertisements, Orders for Copies of The Hospital, and Business Communications should be addressed to THE MANAGER (Not to the Editor), The Hospital, The Hospital Building, 28 & 29, Southampton Street, Strand, London, W.C.**

**The Cost of Subscription, post free, is as follows:—Per week, 2½d.; per quarter, 2s. 8d.; per half-year, 5s. 3d.; per annum, 10s. 6d. Foreign:—Per annum, 15s. 2d., payable, in all cases, in advance.**

**NOTICE.—Vol. XXVII. of "The Hospital" (October, 1899, to April, 1900), in handsome cloth binding, is now on sale at the Office of this Journal, price 7s. 6d. Cases for binding the Numbers contained in this Volume 1s. 6d. Index to Vol. XXVII., 2½d., post free.**

**The Monthly Part for June will be ready on June 1. Price 6d., by post 8d.**

### NOTES ON BOOKS.

Dr. Geo. Steele Perkins has published in pamphlet form a recent address on Lateral Curvature of the Spine, in which he gives a good outline of the causes and treatment of this far-too-common affection.

We have received a little pamphlet, entitled Lunacy: The Rising Tide, written by Mr. W. J. Corbet, M.P., with the object of assisting to clear up some of the doubt which hangs over the question whether the increase of lunacy is real or only apparent; in doing which, however, he does not seem to be very successful.

As a study in good works let us commend a handsome volume, entitled Passmore Edwards Institutions, by J. J. Macdonald (Strand Newspaper Company), which gives an interesting account of the many institutional buildings provided by Mr. Passmore Edwards during recent years. We are told not to hide our light under a bushel, and the publication of this book not only prevents such a catastrophe, but may serve as a guiding light, leading others to follow Mr. Passmore Edwards' good example.

### BOOKS RECEIVED.

Baillière, Tindall, and Cox.

"Diseases of the Gall-Bladder and Bile-Ducts, including Gall Stone." By A. Mayo Robson, F.R.C.S.

Smith, Elder, and Co.

"Paralytic Deformities of the Lower Extremities." By E. Noble Smith, F.R.C.S.Edin., L.R.C.P.Lond.

Accouchement Requisites.

Ambulance Cases.

### NOW READY,

A REPRINT OF

### A NURSING HANDBOOK

TOGETHER WITH S. MAW, SON, AND THOMPSON'S

### COMPLETE CATALOGUE OF NURSING REQUISITES.

The above work is now republished at a cost of 1s. per copy. Messrs. S. M., S., and T. have been compelled to make this charge on account of the extreme popularity of the work and the great expense incurred in its production.

***Post Free on receipt of 1s.***

### S. MAW, SON, and THOMPSON,

7 to 12, ALDERSGATE STREET, LONDON, E.C.

